# Peer-delivered systems-integrated suicide prevention for women in Pakistan: a protocol for hybrid type II pilot cluster randomized controlled trial

**DOI:** 10.1186/s40814-026-01841-7

**Published:** 2026-06-13

**Authors:** Javeria Tanveer, Erum Hamid, Gul Saeed, Sidra Mumtaz, Muhammad Ahmad, Sundus Rahman, Noor-ul-Huda, Shumaila Saleem Malik, Nikita Rao, Atif Rahman, Abid Malik, Siham Sikander, Ashley Hagaman

**Affiliations:** 1 Human Development Research Foundation, Islamabad, Pakistan; 2 Department of Social and Behavioral Sciences, Yale School of Public Health, Yale University, New Haven, CT USA; 3 Department of Biostatistics, Yale School of Public Health, Yale University, New Haven, CT USA; 4 Department of Primary Care and Mental Health, Institute of Population Health, University of Liverpool, Liverpool, UK; 5 Health Services Academy, Islamabad, Pakistan

**Keywords:** Suicide prevention, Maternal health, Implementation science, LMIC, Peer interventions, Health systems, Pilot randomized controlled trial, Pakistan

## Abstract

**Background:**

South Asia has the highest rates of suicide fatalities among women globally. Existing suicide prevention interventions are largely based on Western-informed risk reduction programs and theories. This pilot trial aims to test the acceptability and feasibility of a co-designed suicide prevention program for peri-rural Pakistani women called the Khushal Pur-Umeed Zindagi (KPZ, خوشحال پُرامید زندگی پروگرام), compared to enhanced usual care (EUC). The study seeks to innovate suicide prevention by grounding interventions in decolonized frameworks, leveraging peer mothers, and developing robust health system integration strategies.

**Methods:**

We will conduct a two-arm, mixed-methods, hybrid type 2, stratified pilot cluster randomized controlled trial in peri-rural areas of the Islamabad Capital Territory (ICT). KPZ is a decolonized, culturally salient brief intervention combining narrative-based safety planning and contact follow-up, delivered by peer mothers over 6 months. EUC entails the WHO *Mental Health Gap Action Programme* (mhGAP) suicide prevention module, along with referral support delivered through Primary Health Care (PHC) by Medical Officers (*n* = 2) and Lady Health Worker teams (*n* = 11). KPZ is integrated into the PHC system and is implemented through close collaboration between peer mothers (delivery agents) and the government-employed LHWs. A cohort of peer mothers (*n* = 11), identified from the same communities as participants, will be trained and supervised for the study duration. We will enroll 50 women aged 18–45 with a child under 3 years who report suicidal ideation and will conduct follow-ups at 3- and 6-months post-recruitment. Qualitative interviews with trial participants and periodic reflections from peer mothers will be conducted iteratively over 6 months. A mixed-methods approach will be used to assess both clinical and implementation outcomes. Primary clinical outcomes include suicidal ideation severity and suicidal behaviors. Implementation outcomes include feasibility, acceptability, fidelity, appropriateness, and peer mothers’ competence. Secondary outcomes assess additional clinical domains (depression, anxiety) and culturally relevant constructs (moral injury, cultural suicide cognitions).

**Discussion:**

This pilot trial will provide evidence on the feasibility and acceptability of integrating a decolonized, peer-delivered suicide prevention program for women into the existing Pakistani health system. The findings have the potential for large-scale public health impact by improving the availability, accessibility, and quality of culturally appropriate suicide prevention interventions in resource-constrained and complex environments. Results will inform a larger definitive trial of a community-initiated suicide prevention program in Pakistan.

**Trial registration:**

NCT06208293

**Supplementary Information:**

The online version contains supplementary material available at 10.1186/s40814-026-01841-7.

## Background

### Burden of suicide in low- and middle-income countries

Suicide remains a major and preventable cause of death worldwide, with an estimated 727,000 deaths and more than 20 million suicide attempts occurring each year [1]. The burden of suicide is borne disproportionately by low- and middle-income countries (LMICs), which accounted for nearly 73% of all global suicide deaths in 2021 [1]. Despite this inequitable distribution, the evidence base for suicide prevention interventions remains heavily skewed toward high-income settings, with relatively few interventions developed, adapted, or rigorously evaluated in LMIC contexts.

Preventing suicide is recognized as a global public health priority and is explicitly embedded within the United Nations Sustainable Development Goals (SDGs) [2]. Suicide mortality rate is captured under Indicator 3.4.2 of SDG 3 (Good Health and Well-being) [2], underscoring international commitments to reduce premature mortality from non-communicable conditions, including suicide. However, progress toward this target has been uneven, particularly in resource-constrained settings where structural inequities, limited mental health infrastructure, and sociocultural barriers continue to constrain effective prevention efforts [3, 4]. This study directly aligns with SDG 3 by addressing the critical gap in contextually grounded, scalable suicide-prevention strategies in LMICs, with the aim of contributing to reductions in suicide-related mortality among populations at heightened risk.

### Burden of maternal suicide in LMICs and Pakistan

Maternal suicide is a critical yet under-recognized contributor to maternal mortality in LMICs. Estimates suggest that up to 20% of maternal deaths in LMIC settings are attributable to suicide, and that as many as 27% of women experience suicidal ideation during the perinatal period [5, 6]. Despite this significant burden, robust surveillance data on maternal suicide remain limited in many resource-constrained LMIC contexts, constraining both accurate estimation and effective prevention planning.

Available evidence nonetheless points to important regional and gendered differences in suicide risk profiles. In high-income countries, suicide mortality rates are consistently higher among men than women, with an approximate male-to-female ratio of 5:3. In contrast, in South Asia this ratio is nearly equal, indicating that women in the region account for a substantially larger proportion of suicide deaths than is observed in high-income settings [7]. This pattern underscores the need for context-specific, gender-responsive suicide prevention strategies.

In Pakistan, the estimated suicide rate in 2019 was 8.9 deaths per 100,000 population [8]. Although suicide was decriminalized in Pakistan in 2022 [9], the extent to which this policy shift has translated into improved reporting, access to care, or changes in social attitudes remains largely unknown. Recent qualitative and mixed-methods research has begun to address these gaps by generating grounded theories that identify key psychosocial and structural targets for suicide prevention among women [10], as well as documenting the intergenerational consequences of maternal suicidality for child mental health and development [11].

Taken together, these findings highlight an urgent need for gender-sensitive, community-anchored, and systems-integrated approaches to suicide prevention that address both individual distress and the broader social and structural determinants shaping maternal suicidality in Pakistan and similar LMIC contexts.

### Suicide prevention interventions in LMICs and the need for task-shifted, decolonized, community-anchored, peer-led, and systems-integrated suicide prevention

To date, there are few community-based suicide prevention interventions for adults in LMICs, and even fewer that explicitly target women, despite women bearing a disproportionate burden of suicide attempts in South Asia. Most existing evidence from LMICs has focused either on clinical populations with recent histories of self-harm or on facility-based detection and referral, rather than on preventive, community-anchored approaches.

In South Asia, one of the most robust adult suicide prevention trials is the culturally adapted manual-assisted problem-solving intervention (C-MAP), which demonstrated feasibility and effectiveness in reducing suicidal ideation among adults with a recent history of self-harm [12]. While C-MAP represents an important advance in culturally responsive intervention development, it targeted individuals already engaged with clinical services following severe self-harm and required delivery by trained mental health professionals [12], limiting its scalability in settings with constrained specialist capacity. At the systems level, the World Health Organization’s (WHO) *Mental Health Gap Action Programme* (mhGAP) includes guidance on the identification and management of self-harm and suicide risk [2], yet implementation has largely relied on generalist clinicians and facility-based care, with limited attention to sustained, community-level prevention or gender-specific needs. mhGAP in Pakistan has not been evaluated for its impact on suicide prevention.

Task-shifting has emerged as a key strategy for reducing the burden of mental health conditions in low-resource contexts [3, 13, 14]. However, important gaps remain in understanding how best to adapt and deliver suicide prevention interventions across both low- and high-resource settings. Strengthening suicide prevention requires moving beyond reliance on clinical specialists and meaningfully involving non-specialist providers for timely detection and intervention.

Although evidence-based practices (EBPs) for suicide prevention exist, little is known about their implementation in contexts with limited access to mental health care. To address this, the WHO has recommended non-specialist, low-cost EBPs, such as the Brief Intervention and Contact (BIC), for suicide prevention in LMICs [15]. BIC has been tested with clinicians in several LMICs, including one hospital in Pakistan, and more recently was combined with safety planning, an approach involving personalized coping strategies and support contacts during suicidal crises, delivered by peers in India [12, 15–17].

Building on these efforts, Pakistan has already demonstrated the potential of task-shifting models. Lay peers (i.e., non-specialists) have been successfully engaged to deliver interventions for maternal depression and related mental health concerns [13, 14, 18, 19]. Importantly, stigma remains one of the most significant barriers to suicide disclosure, detection, and care in South Asia [20–22]. Families often conceal suicidal behavior due to fears of social judgment, loss of honor, and religious condemnation, which contributes to severe under-reporting and delayed help-seeking [23, 24].

Saeed and colleagues’ [10] grounded theory study on maternal suicidality in Pakistan further illuminates the psychosocial mechanisms that interventions should target. Women’s suicidal thoughts and behaviors were largely shaped by social isolation and neglect within the *susraal* (in-laws’ home), psychological distress related to the demands of motherhood, unresolved grief, and diminished *sabr* (patience). Protective factors included religious faith and a sense of duty toward children. These findings highlight the need for interventions that can reduce isolation, normalize conversations about distress, address stigma, and provide culturally sensitive emotional support mechanisms that peer-delivered programs are uniquely positioned to address.

Peers are therefore not only feasible and acceptable delivery agents, particularly among women, but also ideally suited to intervene on the culturally and contextually specific drivers of maternal suicidality identified in this study. By leveraging trust, proximity, and shared cultural experience, peers can help bridge Pakistan’s substantial mental health treatment gap while targeting the most salient mechanisms of risk and protection [24, 25]. Accordingly, this research targets peers to implement a culturally informed suicide prevention intervention.

### Pilot trial rationale

This study seeks to move beyond traditional Western paradigms and center locally grounded, culturally responsive approaches for suicide prevention. Our team sought to co-design a decolonized and community-anchored suicide prevention intervention for women by foregrounding epistemological diversity and leveraging scalable, task-shifted implementation strategies suitable for resource-constrained settings [26]. In Pakistan, community health workers and non-clinical Peers have been successfully engaged in the delivery of evidence-based interventions for maternal depression in close partnership with the health system [14, 27, 28]. Building on this strong implementation foundation, we co-developed a community-based, peer-delivered suicide prevention programme tailored to the sociocultural context of Pakistan through extensive formative research and sustained engagement with individuals and families with lived experience of suicidality [10]. Importantly, this work aligns with global public health priorities articulated in the WHO’s *LIVE LIFE* Initiative, a comprehensive framework guiding countries in scaling up evidence-based suicide prevention strategies [1]. Community-based, peer-delivered interventions directly advance the two focal domains of early identification and support for everyone affected by suicide. Collectively, our approach contributes to global efforts to achieve SDG 3 of reducing suicide mortality by one-third by 2030 [2].

To date, the Khushal Pur-Umeed Zindagi (KPZ, خوشحال پُرامید زندگی پروگرام) program has not been evaluated for its acceptability, feasibility, or implementation potential within community settings in Pakistan. We therefore undertake a pilot randomized controlled trial to assess the acceptability and feasibility of implementing KPZ among married women in the maternal period, defined as those who are currently pregnant or have a child under 3 years of age. This manuscript describes the protocol for the pilot trial of the KPZ programme in peri-rural Pakistan. The specific objectives of the study are outlined below.

### Objectives of the study

1. To evaluate the feasibility of the peer-delivered KPZ intervention for reducing suicidal ideation, behaviours, and attempts among adult married women in the peri-rural areas of Islamabad capital Territory (ICT), Pakistan.
2. To evaluate the preliminary effectiveness of the peer-delivered KPZ intervention in reducing suicidality and improving mental well-being among adult married women, compared with Enhanced Usual Care (EUC), at 3- and 6-months post-recruitment in Pakistan.
3. To assess the acceptability and appropriateness of the KPZ intervention and its implementation among peer mothers and participants.
4. To evaluate the appropriateness of KPZ and trial safety procedures, from the perspectives of married women, peer mothers, trainers, and primary health care providers.
5. To assess treatment adherence among adult married women receiving peer-delivered KPZ intervention sessions.
6. To assess the preliminary fidelity of KPZ intervention implementation.

## Methods and design

### Study setting

This pilot trial will be conducted within the Federal District, ICT of Pakistan, which has a population of approximately 2.2 million. Administratively, ICT is divided into 55 Union Councils (UCs). The trial will take place in two peri-rural UCs—Shah Allah Ditta and Sihala—selected due to the study team’s established engagement with local primary healthcare facilities and the Lady Health Workers (LHWs) Program. Both UCs have economies driven primarily by subsistence farming, low-paying government jobs, and semi-skilled or unskilled labor in nearby cities. The public primary healthcare facilities serving these UCs are a Community Health Centre (CHC) in Shah Allah Ditta and a Rural Health Center (RHC) in Sihala. Each facility serves as the first point of contact for the UC population for primary care and reproductive health services. Staffing at each facility includes a Medical Officer, a midwife, a pharmaceutical dispenser, a vaccinator, and 15–20 LHWs supervised by a Lady Health Supervisor (LHS). Each LHW serves a catchment area of approximately 1200–1700 people (roughly 200–250 households), visiting each household monthly. Two LHWs live within the same communities they serve, and one room of their residence is designated as the “Health House,” where women can safely access basic care. Their responsibilities include providing preventive and promotive maternal, neonatal, and child health services, as well as family planning services, to all registered households in their catchment area.

### Study design

The study is a mixed-methods pilot hybrid type-II stratified cluster randomized controlled trial (cRCT), with six Village Clusters (VCs) allocated in a 1:1 ratio either to the intervention or EUC arm across two UCs (Shah Allah Ditta and Sihala). All study elements were designed alongside a Community Advisory Board (CAB) comprising five women with lived experience of suicidal thoughts or behavior and two LHWs from the study sites. The CAB provided ongoing feedback on all study materials, including recruitment procedures, intervention content, assessment tools, and implementation strategies, ensuring cultural relevance, acceptability, and feasibility. The CAB initially met quarterly during the co-design phase and then annually throughout the study to guide research operations. Recruitment of women of reproductive age is planned to span 1.5 years, with endline follow-ups of all participants 6 months post-recruitment beginning in May 2024.

Mixed-methods include quantitative measures and multiple qualitative methods, including longitudinal periodic reflections, in-depth interviews, focus group discussions, and systematic meeting debriefs to assess implementation outcomes and mechanisms. Longitudinal periodic reflections involve recurring discussions with implementing agents, namely peer mothers, to capture their evolving experiences, challenges, and adaptations over the course of intervention delivery. In-depth interviews with trial mothers, peer mothers, and health system clinicians elicit detailed insights into the feasibility, acceptability, and perceived mechanisms of change, as well as familial, social, and political factors influencing engagement and implementation. Systematic meeting debriefs provide documentation of emerging issues, solutions, and contextual factors that may affect implementation fidelity, reach, and sustainability of the intervention. Together, these qualitative methods allow for a comprehensive understanding of both participant and implementer experiences, inform iterative improvements, and contextualize quantitative outcome measures within the lived realities of the study setting.

### Randomization

The unit of randomization will be the VC. Each VC comprises 2–3 adjoining LHW catchment areas, with a total population of approximately 2400–3600 individuals. Randomizing at the VC level minimizes the risk of contamination between study arms. Six eligible VCs, across two UCs, will be randomized to the intervention or control arms using a 1:1 allocation ratio, with stratification at the UC level. Randomization will occur prior to participant identification and recruitment. Research teams responsible for participant identification, consent, and recruitment will remain blinded to VC allocation to minimize post-randomization recruitment bias.

**Fig. 1:**
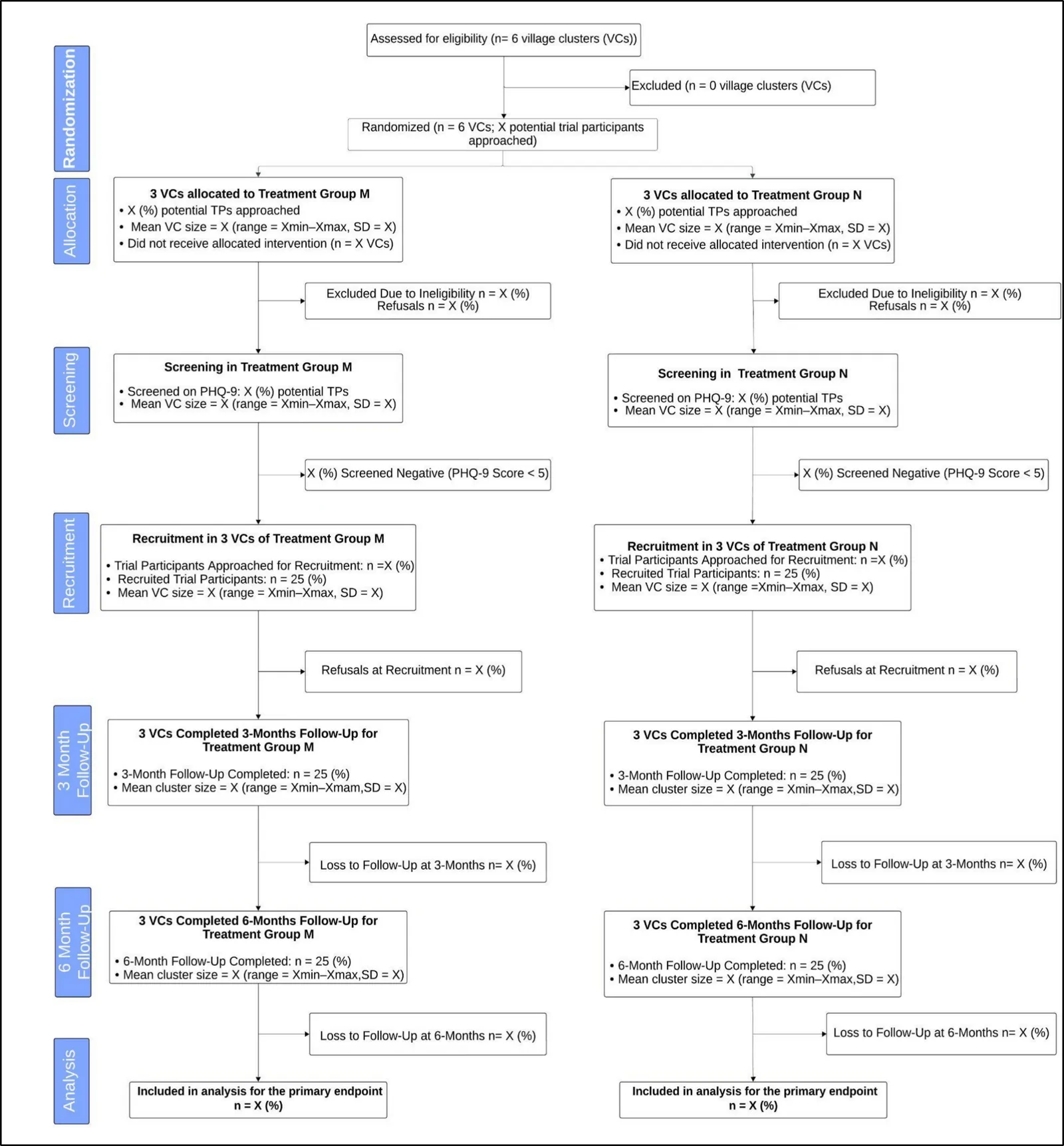
CONSORT Flow Diagram of Detailed Progress of Subjects in the Trial

Stratification at the UC level enhances study precision and statistical power by reducing variability between intervention and control VCs. It also helps prevent imbalances across arms that could result from UC-level factors, such as socioeconomic development, which may influence study outcomes. The randomization process and overall study design are illustrated in a CONSORT flow diagram (Fig. 1), prepared in accordance with the CONSORT extension for pilot cluster randomised trials [29]. VCs will be randomized through a computer-generated program by an individual at Yale University not involved in the day-to-day activities of the trial.

### Ethical approval

Ethical approval will be obtained from the appropriate institutional ethics committee prior to the initiation of the trial. Ethical approval will be obtained from the Ministry of National Health Services, Regulation and Coordination (Pak Secretariat, Islamabad), M/o National Health Services Regulation and Coordination (District Health Office-ICT), National Bioethics Committee (NBC) and Yale University.

## Eligibility, sampling, recruitment, enrollment, and consent procedures

### Eligibility

#### Inclusion criteria

Women of reproductive age (between 18 and 45 years) are either pregnant or have a child under 3 years; report a positive response to item 9 of the Urdu Patient Health Questionnaire (PHQ-9); can speak Urdu fluently; have access to a mobile phone; are connected to primary health care services; and intend to live in the study area for at least 6 months.

#### Exclusion criteria

Women requiring immediate inpatient care for medical or psychiatric conditions, no access to a mobile phone, and unable to speak and understand Urdu.

#### Sampling procedures

The trial aims to recruit a total of 50 participants at risk of suicide and allocated into two equal groups: an intervention arm (*n* = 25) and an EUC arm (*n* = 25) based on their cluster randomization. This sample size is consistent with recommended guidelines for pilot randomized-controlled trials [30] and is sufficient for the primary goal of estimating key feasibility parameters, including recruitment rates, retention, and preliminary effect sizes, to inform the design of a future definitive trial, rather than for testing statistical significance. Participants will be identified by screening for suicidal ideation (in partnership with the local government health system) across six VCs. Based on extant literature and preliminary formative data, which estimate a 5–8% prevalence rate, we will screen approximately 800 women to ensure adequate recruitment across this range. The target analytic sample for all subsequent 3- and 6-month follow-up assessments will remain 50 participants, with robust retention strategies employed to minimize attrition. We will also aim to recruit 12 peer mothers to be trained as delivery agents for the KPZ intervention.

### Recruitment procedures

#### Screening

Screening procedures were co-developed with the CAB, LHWs, and the local government health system. Women of reproductive age identified by LHWs within their catchment areas across Shah Allah Ditta and Sihala UCs will be approached for screening by LHWs who then introduce trained members of the research team. Screening will assess recent suicidal ideation and eligibility for study participation. Women who meet eligibility criteria will be invited to participate in the trial.

### Recruitment

During the co-design phase of the KPZ intervention, LHWs were oriented to the study objectives and trained to sensitively approach women experiencing suicidal distress and their families within their catchment areas. Women who report suicidal ideation within the past 2 weeks and meet eligibility criteria will be recruited into the study following informed consent obtained by the research team. Enrolled participants will be followed up at 3- and 6-months post-recruitment. Participants in the intervention arm will receive the peer-delivered intervention. Participants in both study arms will be referred to the Medical Officer (MO) at the respective primary healthcare facility, who is trained in the WHO mhGAP including identifying and managing common mental conditions and suicide. If imminent suicide risk is identified, the research team will negotiate with the participant and her family to refer her to specialized psychiatric care at the nearest hospital. Participants allocated to the control arm will receive EUC, with the same referral and safety procedures applied in cases of active suicidal ideation. Written informed consent will be obtained from all participants in both study arms prior to enrollment.

### Informed consent procedure

Careful attention to informed consent and other study safety procedures was co-designed with the CAB to optimize salience, understanding, and collective safety. Trained research assistants will obtain written informed consent from all participants prior to any study procedures. The consent form will be read aloud to participants, and, with the participant’s permission, one or more trusted household members may be present during the consent process to support understanding and decision-making. Informed consent will be obtained prior to screening and baseline interviews, with consent reaffirmed verbally at the 3- and 6-month follow-up assessments. All interviews will be conducted at locations preferred by participants, in their homes, the LHW Health House, or another preferred location.

Participants will be informed that all personal information will be kept confidential, that study data will be anonymized and stored securely in locked cabinets and password-protected systems, and that data will be used solely for research purposes. Participants will also be informed of the specific circumstances under which confidentiality may be breached to protect their safety or the safety of others, as well as the procedures that will be followed in such cases. As part of the consent process, participants will be asked to identify an emergency contact in the event that a confidentiality breach is required. Where appropriate and with participant consent, family members may be engaged as part of safety planning to support participant well-being. Scripts for engaging and negotiating with family members were designed by the CAB.

Further, potential peer mothers will be identified by LHWs within their respective VC/UCs. An orientation meeting will be conducted with eligible candidates at their respective primary healthcare facilities, during which the goals of the KPZ intervention and the responsibilities of the peer mother role will be explained in detail. Women who express interest and meet eligibility criteria will be invited to participate and written informed consent will be obtained prior to their recruitment. Formal engagement of peer mothers will be completed through written agreements outlining their roles, responsibilities, and ethical obligations within the KPZ intervention.

### Refusal to consent

Participation in the study will be entirely voluntary. Participants will be able to withdraw their consent from any part of the study at any time, including the intervention and/or follow-up, without needing to provide a reason.

## Assessments

### Baseline assessment

All baseline assessments will be conducted by trained members of the research team who are blinded to participants’ allocation status. Baseline data will include sociodemographic characteristics, including age, marital status, reproductive history (including previous pregnancies and miscarriages), educational attainment, and employment status.

Baseline assessments will also measure the severity of suicidal ideation, depression, and anxiety; perceived social support; suicide-related cognitions; exposure to intimate partner violence (IPV); moral injury; and health services utilization in the 6 months preceding enrollment. Detailed descriptions of the assessment instruments used for each construct are provided in Table 1.

**Table 1:** Primary and secondary outcome measures

Primary outcome measures		
Outcome measure	Measure description	Time frame
Beck's Scale for Suicidal Ideation (BSSI)	Suicidal ideation severity will be measured using the Urdu validated BSSI [34, 35]. The BSSI is a 19-item self-report instrument that assesses the intensity of suicidal thoughts, attitudes, and behaviors over the past 2 weeks. Each item is rated on a 3-point scale (0–2), with total scores ranging from 0 to 38; higher scores indicate greater severity of suicidal ideation	baseline and 6 months post-enrollment
Suicidal behaviors measured by Columbia Suicide Severity Rating Scale (C-SSRS)	Suicidal behaviors and attempts will be assessed using the C-SSRS [36]. The C-SSRS captures self-reported suicide attempts, aborted attempts, and interrupted attempts using dichotomous (yes/no) items. Any endorsement of these behaviors will be coded as a positive suicidal behavior outcome	baseline and 6 months post-enrollment
Secondary outcome measures		
Outcome measure	Measure description	Time frame
Patient Health Questionnaire—9 item (PHQ-9)	Depressive symptoms will be measured using the Urdu validated PHQ-9 [37], a 9-item self-report instrument assessing the frequency of depressive symptoms over the past 2 weeks. Items are rated on a 4-point scale ranging from 0 (not at all) to 3 (nearly every day), with total scores ranging from 0 to 27; higher scores indicate greater severity of depressive symptoms	Baseline, 3 and 6 months post-enrollment
Generalized Anxiety Disorder—7 item (GAD-7)	Anxiety will be measured using the Urdu validated GAD-7 [38], a 7-item self-report instrument. Items are scored on a 4-point scale from 0 (not at all) to 3 (nearly every day), with total scores ranging 0–21: higher scores indicate greater severity of anxiety symptoms	Baseline, 3 and 6 months post-enrollment
Multidimensional Scale of Perceived Social Support (MSPSS)	Social support will be measured using the Urdu-validated 12-item Multidimensional Scale of Perceived Social Support [39]. The MSPSS assesses perceived social support from three sources: family, friends, and significant others. Respondents rate each item on a 6-point Likert scale (0 = strongly disagree to 5 = strongly agree), with higher scores indicating greater perceived support	Baseline, 3 and 6 months post-enrollment
Maternal Suicide Risk Inventory (MSRI)	We developed this measure from formative interviews with 20 women with a history of suicidal behavior or clinical practice with suicidal women and 3 focus group discussions with Pakistani clinicians to identify inductively defined mechanisms of suicidality. The items will assess cognitive and emotional characteristics hypothesized to lie on the pathway to suicidality (e.g., loneliness, abandonment, helplessness, hopelessness, perceived burdensomeness, anger, and patience). Items are rated on a 3-point Likert scale and summed to yield total scores ranging from 16 to 48, with higher scores indicating greater suicide-related risk cognitions	Baseline, 3 and 6 months post-enrollment

### Follow-up assessments

All participants will be followed up at 3- and 6-months post-recruitment by trained research staff blinded to allocation status of the participants. Follow-up assessments will reassess suicidal ideation, depression, anxiety, perceived social support, suicide-related cognitions, intimate partner violence, moral injury, and health services utilization since the previous assessment. A subsample of purposively selected trial participants will be approached at 1 month and/or 6-months post-enrollment to qualitatively elicit experiences related to their suicidality, the trial, and the health system.

## Intervention groups

### EUC arm

Participants allocated to the control arm will receive EUC. Under this condition, LHWs will link women identified as at risk of suicide to their primary care facility-based MO. The MOs will be trained on the WHO mhGAP suicide prevention and risk assessment module [16]. In cases of imminent or lower-risk suicidality, LHWs will ensure participant’s immediate safety and facilitate a visit to the MO or higher-level specialty care as appropriate. In addition, all participants and healthcare workers will be provided with a nationally available suicide crisis hotline number supported by Taskeen, a Pakistani mental health organization.

### KPZ intervention arm

Participants allocated to the intervention arm will receive a total of 12 sessions delivered by a trained and supervised lay peer mother. KPZ comprises co-designed, brief suicide prevention interventions, including a decolonized approach to crisis and safety planning [31, 32], followed by structured follow-up sessions. These sessions explicitly target mechanisms identified in the grounded theory of maternal suicidality, including strengthening social connectedness, fostering *sabr* (patience), negotiating family and interpersonal stressors, and supporting the implementation of individualized coping and help-seeking strategies.

The decolonized safety planning component adapts conventional safety planning to enhance cultural relevance by reconstructing key domains to reflect mothers’ lived realities, employing a storytelling-based delivery approach, and incorporating visual illustrations created by Pakistani artists to convey the purpose and meaning of each step. Peer mothers will be trained in basic psychosocial support and in delivering all session content with fidelity.

All participants in the intervention arm will receive a KPZ booklet that accompanies each session. The booklet centers on the evolving story of a fictional character, Shagufta, whose experiences reflect common maternal stressors in the study context, allowing participants to relate their own life circumstances to the narrative. The booklet is primarily image-based rather than text-based to ensure accessibility for participants with limited literacy.

The 12 sessions will be delivered over a 6-month period. The first five sessions will occur within the first month, sessions 6 through 9 will be conducted biweekly, and sessions 10 through 12 will be conducted monthly. LHWs supporting participants in the intervention arm will follow the same safety monitoring procedures and referral pathways to MOs and higher-level care as outlined for the EUC arm.

## Intervention discontinuation and participant discontinuation criteria intervention will be discontinued if trial participants develop psychosis or another severe mental disorder.

### Loss to follow-up

Any participant who cannot be followed up at the designated time points (i.e., 3 and 6 months postnatal) will be considered lost to follow up and not contactable by the research team. Those who wish to withdraw consent from further intervention sessions; it will be recorded, and they will be asked if they wish to be followed-up or not, which will also be recorded. In case of consent withdrawal at any follow up, data collected up to that point of withdrawal will be included in the analyses.

### Masking

Due to the nature of the intervention, it is not possible to mask participants and delivery agents to the treatment allocation. However, outcome assessments will be masked/blinded, as all outcome measures will be administered by trained research assessment teams, who will be non-residents of the study area, independent of the intervention and blinded to treatment allocation of the participants. Furthermore, participants will be instructed not to disclose any information about the intervention or the delivery agent assessment team. The intervention implementation team and outcome assessment teams will not have any interactions during the trial since there will be separate locations and administrative management.

## Monitoring for adverse events

### Serious adverse events (SAEs)

A serious adverse event (SAE) is a serious medical or psychological issue that happens to a participant, even if it is not caused by the study. The following events will be considered potential serious adverse events (SAEs) and will be reported accordingly. This list is not restrictive; any incident reported will be assessed against the defined seriousness criteria and reported if deemed appropriate. Potential SAEs include (1) death of a participant from any cause; (2) any suicide attempt; (3) observed incidents of domestic violence or victimization involving the participant; (4) hospital admission due to psychiatric issues; (5) hospital admission due to a serious medical emergency; (6) miscarriage, stillbirth, or loss of a child; and (7) any other event that meets the seriousness criteria as outlined in the protocol (or is otherwise deemed medically significant by the site investigator).

The reported adverse event will be assessed for seriousness by an independent psychiatrist based at the site, who will evaluate the event, determine its relevance to the trial, and advise on any necessary actions. If the event is judged to be related to the trial. 

Principal Investigator (PI) will report it to both the relevant ethics committees and the Data and Safety Monitoring Board (DSMB) within 72 h. Additionally, all SAEs regardless of relatedness will be compiled and reported in a blinded format every 6 months to both the Yale Institutional Review Board (IRB) and the DSMB.

### Other adverse events (OAEs)

In addition to the SAEs, other adverse events (OAEs) will be reported to the Institutional Review Board (IRB). This includes (1) any serious or non-serious adverse event that is unanticipated and indicates that the research places participants or others at greater risk of harm than was previously known or recognized, (2) information that indicates an adverse change to the risks or potential benefits of the research or allegation of non-compliance with protocol requirements, (3) breach of confidentiality, (4) incarceration of a participant in a protocol not approved to enroll prisoners, (5) sponsor-imposed suspension for risk, (6) complaint of a participant if it indicates unexpected risks or cannot be resolved by the research team, (7) any protocol deviation or violation that harmed participants or others, (8) that indicates participants or others may be at increased risk of harm, (9) or that compromises the integrity of the research data, (10) any protocol changes made without prior IRB approval to eliminate apparent immediate harm and any safety reporting requirements specified by the IRB as a condition of approval.

Other Adverse Events (OAEs) will be documented if identified by the delivery agents—peers in the intervention arm or Lady Health Workers (LHWs) in the Enhanced Usual Care (EUC) arm—or by peer supervisors, and will be addressed. The research team will follow the guidelines of the Yale University IRB and the National Bioethics Committee of Pakistan regarding unanticipated problems that pose risks to participants or others.

### Training and supervision of peer mothers

Peer mothers’ selection was co-developed in collaboration with the CAB and informed by the research team’s extensive experience implementing task-shifting mental health interventions in Pakistan. Peer mothers will be lay women with no formal mental health training who have demonstrated an interest in supporting other women in their communities. Eligibility criteria include being aged 18 years or older, regardless of marital status; fluency in Urdu; completion of at least matriculation education (Grade 10); residence in the same or nearby communities as trial participants; and an intention to remain in the study area for the full duration of the follow-up period. Peer mothers will complete a three-phased training and supervision process. Phase 1 will consist of a 4-day, in-person training delivered by Pakistani female Peer Clinical Psychology Supervisors with master’s-level training in psychology. This phase emphasizes interactive learning approaches, including group discussions and role-playing exercises, and covers core content areas such as the KPZ intervention components, ethical conduct and confidentiality, managing difficult or distressing situations, and the identification, documentation, and reporting of serious adverse events (SAEs).

Phase 2 will involve 6–7 weeks of supervised field-based training, during which peer mothers deliver KPZ sessions to practice cases. All sessions will be either directly observed or audio-recorded and reviewed by peer supervisors. During this phase, peer mothers will participate in weekly one-on-one supervision meetings focused on structured feedback, skills development, and competency assessment.

Phase 3 will include ongoing group-based supportive supervision with all peer mothers throughout the intervention period. Supervision is grounded in a supportive supervision model and focuses on addressing implementation challenges through role-play and problem-solving, reinforcing effective practices, maintaining peer mothers’ motivation and well-being, and conducting periodic competency assessments to ensure fidelity and quality of delivery.

### Evaluating competency of peer mothers delivering KPZ

Upon completion of training, peer mothers will undergo competency assessments using structured, standardized role-playing exercises. The 19-item Enhancing Assessment of Common Therapeutic Factors tool ENACT [33] will be used to assess peer mother competence at the initial stage (baseline) of KPZ delivery. A minimum score of 70% is required for a peer mother to qualify to deliver the intervention. Peer mothers will be re-assessed at midline and endline during monthly supervision sessions through role-plays based on scripted scenarios. Only those peer mothers who consistently meet the required competency levels will be selected to deliver KPZ in the trial. During assessments, trainers will conduct one-on-one role-plays with each peer mother while the remaining peer mothers observe. Peer mothers will first reflect on their own performance, followed by detailed feedback from trainers. If a peer mother scores below the required competency threshold at any point, she will receive refresher training to regain the necessary competency before proceeding.

## Measures

Table 1 below summarizes all primary and secondary outcome measures and Table 2 below summarizes all implementation outcome measures, including their descriptions and assessment time points.

**Table 2:** Implementation outcome measures

Implementation outcomes		
Outcome measure	Measure description	Time frame
Trial feasibility		
Percent eligible participants who consent	Proportion of individuals who met eligibility criteria and provided informed consent to participate in the study	At recruitment
Percent of consented participants who started intervention	Proportion of participants who initiated at least one KPZ intervention session after consenting	6 months
Percent of participants who completed KPZ safety card	Proportion of enrolled participants who completed the KPZ safety card component	6 months
Percent of participants who completed one brief contact follow up session	Proportion of enrolled participants who completed at least one brief follow-up contact session	6 months
Percent of participants who dropped out of KPZ intervention	Proportion of participants who discontinued participation prior to completing the intervention	3 months and 6 months
Fidelity		
Median number of sessions completed	Median number of sessions completed by participants	6 months
Feasibility		
Feasibility of Implementation Measure (FIM)	FIM is a 4-item feasibility scale [41]. The scale was culturally adapted to fit the study and Urdu language	6 months post-enrollment
Acceptability		
Acceptability of Implementation Measure (AIM)	AIM is a 4-item Acceptability scale to assess perspectives on implementation [41]. The scale was culturally adapted to fit the study and Urdu language	6 months post-enrollment
Appropriateness		
Intervention Appropriateness Measure (IAM)	IAM is a 4-item appropriateness scale [41]. The scale was culturally adapted to fit the study and Urdu language	6 months post-enrollment
Cross cutting across FIM/AIM/IAM		
Individual qualitative interviews with intervention mothers	We will conduct qualitative interviews with trial participants to explore their perspectives on the intervention’s content and delivery, aiming to elicit insights into its feasibility, acceptability, and perceived mechanisms of change.”	Baseline and 6 months post-enrolment
Longitudinal periodic reflections with peer mothers and peer supervisors	We will conduct periodic reflections with peer mothers and peer supervisors to regularly check in on the progress of P-SuPP implementation. These reflections will provide space to discuss, document, and reflect on important events, activities, and changes occurring throughout the course of implementation	two elicitations per peer mother, one in first half and one in second half of implementation
Knowledge, attitudes, self-efficacy		
Knowledge, Attitudes, and Confidence (KAC)	The KAC is a 30-item questionnaire developed from our formative co-design research that assesses implementing agent (e.g., peer mother) implementation domains of confidence (11 items), attitudes (acceptability, appropriateness, perceived benefit) (13 items), and knowledge (6 items) in implementing the elements of KPZ with the mother directly and within her social milieu (e.g., family engagement). Items will be responded to with a 4-item Likert scale with responses ranging from 'none of the time' to 'all of the time'. Higher scores indicate more confidence, more positive attitudes, and more knowledge of KPZ intervention components and suicide myths	6 months post- enrollment

### Primary clinical outcomes

Suicidal ideation severity will be assessed using the Beck Scale for Suicidal Ideation (BSSI) [34, 35], while the presence of any suicidal behaviors and attempts will be measured using the Columbia Suicide Severity Rating Scale (C-SSRS) [36]. Both will be administered at baseline and 6-month post-recruitment.

### Secondary clinical outcomes

Depressive symptoms will be measured using the Urdu-validated Patient Health Questionnaire-9 (PHQ-9) [37], while anxiety symptoms will be assessed using the Urdu-validated Generalized Anxiety Disorder-7 (GAD-7) [38] administered at baseline, 3 months, and 6 months.

Perceived social support will be assessed using the Multidimensional Scale of Perceived Social Support (MSPSS) [39]. Suicide-related cognitions will be measured using the Maternal Suicide Risk Inventory (MSRI) [40], a locally developed 16-item measure capturing inductively derived cognitive pathways to maternal suicidality. These two measures will be administered at baseline, 3 months, and 6 months.

### Implementation outcomes

Adoption, reach, fidelity, feasibility, acceptability, and appropriateness will be assessed using a combination of quantitative indicators (e.g., participation, completion, number of KPZ sessions delivered) and validated implementation scales (AIM, IAM, FIM), alongside qualitative interviews, focus group discussions, and longitudinal reflections with peer mothers, supervisors, LHWs, and medical officers. To understand how well the KPZ intervention was received, a combination of quantitative tools and qualitative methods will be used to explore its relevance, appropriateness, and overall acceptability from the perspectives of those implementing and engaging with it.

Three brief standardized scales, the Feasibility of Intervention Measure (FIM), Acceptability of Intervention Measure (AIM), and Intervention Appropriateness Measure (IAM), will be used to assess how implementing agents perceived the intervention [41]. Each scale contains four items rated on a 5-point Likert scale (from “completely disagree” to “completely agree”). FIM will measure how practical and easy the intervention was to deliver. AIM will assess whether the implementers found the intervention appealing and welcomed the approach. IAM will assess how well the intervention seemed to fit the context and needs of the population. Together, these tools provide a quick yet meaningful snapshot of implementers’ views on the intervention’s usability and relevance.

Finally, peer mother knowledge, attitudes, and confidence to implement KPZ will be assessed using the 30-item Knowledge, Attitudes, and Confidence (KAC) scale (Table 3) [42].

**Table 3:** A priori progression criteria for a definitive full-scale trial

	Go-proceed with RCT	Amend-proceed with changes	Stop-do not proceed unless
1.Feasibility of participant recruitment Can 50 trial participants (Assuming each village cluster can recruit 8 participants) be recruited to take part in the study?	If at least 38 trial participants Could be recruited to take part in the study	If < 38 participants are recruited To take part in the study	If < 20 participants are recruited To take part in the study
2. Feasibility of data completion Can at least 70% of recruited participants be retained in the study until completion?	If 70% of recruited participants Are retained in the study until completion of trial	If 70–60% of recruited Participants are retained in the study until completion of trial	If less than 60% of recruited Participants are retained in the study until completion of trial
3.Feasibility of treatment adherence Can t least 70% of participants complete all the 12 sessions of KPZ intervention?	If 70% of recruited participants complete all the 12 sessions of KPZ intervention	If 50–70% of recruited Participants complete all the 12 sessions of KPZ intervention	If less than 50% of recruited Participants complete all the 12 sessions of KPZ intervention

## Qualitative data collection

Qualitative data will be gathered through 8–12 interviews with trial women at risk of suicide; 8–12 interviews with EUC women; and 2 periodic reflections per peer mother. In the intervention arm, trial participants’ interviews will be conducted at two time points, with some interviewed around 1-month post enrollment and others at endline to explore whether there was any change in participant’s view with time/or as the trial progressed. For the EUC arm, women’s interviews will be conducted at endline to explore any facilitators and barriers to receiving referred care, recommendations on what would help suicidal mothers, and experiences with BHU/referral services. For each peer mother, two PR will be completed, one in the first half of implementation and the other in the second half. The qualitative data will explore experiences of the intervention/trial, the course of their suicidality over the 6 months, how the trial/intervention was received/delivered, which aspects worked well or need improvement, and which contextual factors (such as family dynamics, social norms, or health system infrastructure) influenced implementation. Data will be collected in audio recordings (when consented) and detailed field-note summaries that include verbatim quotes. Leveraging template analysis as outlined by [43], findings will be organized into matrices, one for each category of participants, according to the themes that emerged from the data, and the analysis was conducted using these matrices.

### Quantitative analysis

Data will be analyzed using Stata [44] and R [45], following CONSORT guidelines relevant extensions for cluster-randomized and pilot-feasibility trials. Participant flow will be presented using CONSORT diagrams, including numbers screened, eligible, randomized, and analyzed for the primary outcomes. All clinical and feasibility analyses will be conducted based on the intention-to-treat (ITT) principle, where all randomized participants will be included in the analysis in the group to which they were originally assigned, regardless of the treatment they actually received or any protocol deviations. For continuous measures, means (standard deviations) or medians (interquartile ranges) will be reported as appropriate; for categorical outcomes, counts and proportions will be presented.

At baseline, demographic characteristics will be summarized by study arm to assess randomization balance and to facilitate comparison of the distribution of potential confounding factors. For follow-up assessment at 3 and 6 months, clinical outcome measures will likewise be reported by trial arm using summary statistics including means, standard deviations, and interquartile ranges. This consistent reporting structure allows for arm-wise evaluation of outcomes across all time points.

Means and frequencies will be used to describe baseline characteristics, participation rates in the study procedures, instrument/item response rates, and engagement with the peer-mother-delivered intervention. Means and frequencies for each suicide-related outcome (e.g., CSSR-S and BSSI) and secondary outcomes (e.g., PHQ-9, GAD-7) will also be calculated at each assessment point.

Primary and secondary clinical outcomes will be analyzed using mixed-effects models to account for clustering within villages and repeated measures within participants. Continuous outcomes will be analysed using linear mixed-effect models, and binary outcomes using generalized linear mixed-effects models with a logit link, each including fixed effects for study arm, time, and their interaction, and random intercepts for village and participant. Missing data will be handled using these linear mixed-effects models, which account for missingness under the missing at random (MAR) assumption by utilizing all available data points for each participant. Analyses are exploratory and will emphasize effect size estimation and 95% confidence intervals, rather than statistical significance, consistent with the pilot design. To ensure the coverage of confidence intervals is accurate, the Kenward-Roger degrees of freedom correction will be applied to all mixed-effects models. This method specifically corrects both the degrees of freedom and the standard errors to account for the uncertainty in variance component estimation that arises with few clusters, and is expected to produce wider, more conservative confidence intervals. Furthermore, if the model encounters a singular fit due to limited cluster-level information, Bootstrap confidence intervals (percentile method, 1000 iterations) will be utilized as a robust sensitivity analysis, as this does not rely on variance component estimation.

### Other endpoints and exploratory analysis

Feasibility, acceptability, and fidelity endpoints: these outcomes (e.g., retention, session adherence, acceptability, and fidelity measures) will be summarized primarily using descriptive statistics.

### Reporting

Results will be reported in accordance with CONSORT (cluster and pilot/feasibility extensions) and relevant reporting guidance for implementation outcomes in pilot Hybrid Type II designs.

### Power calculation

As outlined by biostatisticians Thabane and Leon, pilot trials are not powered for statistical analysis [46, 47], but data will be monitored regularly. This study is not designed as a definitive, fully powered cRCT but rather as a feasibility/pilot trial intended to inform the power and sample size calculations for a future definitive cRCT and to estimate the Intra-cluster Correlation Coefficient (ICC) for our primary outcomes of interest. Our randomization strategy involves stratification at the UC level, with village clusters randomized 1:1 within each Union Council, to improve the precision of our estimates and provide critical data for the design of the future trial.

## Trial monitoring

An independent Data Safety and Monitoring Board (DSMB) will oversee this study. Prior to enrollment, the DSMB will review the protocol to confirm that appropriate participant safety procedures are in place. The DSMB will include experts in suicide prevention, the Pakistani mental health and health system context, and biostatistics, all of whom will declare no conflicts of interest. The DSMB will monitor study progress, including staff responses to adverse events, protocol adherence, and whether enrollment and data collection are sufficient to address the primary study aims. The Principal Investigator (PI) will lead investigations of adverse events and serious adverse events, with co-investigators assuming this role if the PI is unavailable. Serious adverse events will be reported to the ethical review bodies, DSMB, and NIH within 72 h. Any DSMB-recommended protocol changes or suspensions will be communicated to NIMH within 72 h, along with relevant reports and supporting documentation.

## Dissemination

The findings of this pilot clinical feasibility trial will be disseminated through multiple channels to ensure broad accessibility and impact. The findings will be submitted for publication in peer-reviewed journals regardless of whether the results are positive, negative, or inconclusive. In addition, results will be presented at relevant national and international conferences, and academic meetings.

A summary of the study outcomes will also be made available to study participants upon request, in plain language, to support transparency and participant engagement. Where appropriate, findings will be shared with advocacy groups, healthcare providers, and relevant stakeholders to inform future clinical practice or the design of a definitive trial. The dissemination strategy adheres to the CONSORT extension for pilot and feasibility trials to ensure complete and transparent reporting.

## Discussion

The KPZ peer-delivered pilot trial is designed to address a critically underexplored area of maternal mental health by examining the feasibility and acceptability of a decolonized, community-informed, peer-delivered suicide prevention intervention in a low-resource setting in Pakistan. Situated in peri-rural Pakistan, the study seeks to respond to the persistent neglect of maternal suicidality in both research and practice and to generate contextually grounded evidence to inform future intervention development. As a protocol, this trial focuses on implementation processes rather than clinical effectiveness.

If found feasible and acceptable, the KPZ trial may contribute to the limited evidence base on peer-delivered suicide prevention interventions in LMICs. The study is designed to generate implementation-focused insights relevant to settings with constrained specialist resources and substantial mental health treatment gaps. Features such as structured peer mother training and supervision, as well as mhGAP training for MOs are intended to inform considerations of scalability and sustainability.

More broadly, KPZ is intended to inform global mental health approaches that prioritize cultural adaptation, community engagement, and decolonized practices in intervention design. By documenting the processes through which community-informed suicide prevention strategies can be developed and implemented, the study may offer methodological guidance for future research in culturally diverse and resource-limited contexts.

### Limitations

As a feasibility and acceptability trial, KPZ is not designed to assess clinical effectiveness or suicide-related outcomes. The findings will therefore be limited to implementation processes, acceptability, and feasibility indicators. Nonetheless, the trial is intended to provide essential groundwork for subsequent effectiveness studies by assessing whether a peer-delivered, decolonized suicide prevention approach can be implemented within maternal health contexts in Pakistan.

## Trial status

The pilot trial is currently active and ongoing.

## Supplementary Information

Supplementary Material 1.

## Data Availability

Data and materials will be available from the principal investigator.

## Abbreviations

AIM: Acceptability of Intervention Measure
BIC: Brief Intervention and Contact
BSSI: Beck Scale for Suicidal Ideation
CAB: Community Advisory Board
CHC: Community Health Center
C-MAP: Culturally Adapted Manual-Assisted Problem-solving intervention
cRCT: Cluster Randomized Controlled Trial
C-SSRS: Columbia Suicide Severity Rating Scale
DSMB: Data Safety and Monitoring Board
EBPs: Evidence-based practices
EUC: Enhanced Usual Care
FIM: Feasibility of Intervention Measure
GAD‑7: Generalized Anxiety Disorder 7‑item scale
IAM: Intervention Appropriateness Measure
ICT: Islamabad Capital Territory
KAC: Knowledge Attitudes, and Confidence scale
KPZ: Khushal Pur-Umeed Zindagi intervention
LHWs: Lady Health Workers
LMICs: Low- and middle-income countries
mhGAP: Mental Health Gap Action Programme
MO: Medical Officer
MSPSS: Multidimensional Scale of Perceived Social Support
MSRI: Maternal Suicide Risk Inventory
NBC: National Bioethics Committee
PHQ-9: Patient Health Questionnaire 9-item scale
RHC: Rural Health Center
SAEs: Serious adverse events
SDGs: Sustainable Development Goals
UCs: Union Councils
VCs: Village clusters
WHO: World Health Organization
